# Repurposing of metformin and colchicine reveals differential modulation of acute and chronic kidney injury

**DOI:** 10.1038/s41598-020-78936-5

**Published:** 2020-12-15

**Authors:** Maryam El-Rashid, Danny Nguyen-Ngo, Nikita Minhas, Daniel N. Meijles, Jennifer Li, Kedar Ghimire, Sohel Julovi, Natasha M. Rogers

**Affiliations:** 1 Centre for Transplant and Renal Research, Westmead Institute for Medical Research, 176 Hawkesbury Road, Westmead, NSW 2145 Australia; 2 Molecular and Clinical Sciences Research Institute, St George’s University of London, London, UK; 3 Westmead Clinical Medical School, University of Sydney, Camperdown, NSW Australia; 4 Renal Division, Westmead Hospital, Sydney, NSW Australia; 5 Department of Surgery, Thomas E. Starzl Transplantation Institute, University of Pittsburgh School of Medicine, Pittsburgh, PA USA

**Keywords:** Kidney, Kidney diseases, Innate immune cells, Autophagy, Acute kidney injury, Chronic kidney disease, Diseases, Nephrology

## Abstract

Acute kidney injury (AKI) is a major health problem affecting millions of patients globally. There is no effective treatment for AKI and new therapies are urgently needed. Novel drug development, testing and progression to clinical trials is overwhelmingly expensive. Drug repurposing is a more cost-effective measure. We identified 2 commonly used drugs (colchicine and metformin) that alter inflammatory cell function and signalling pathways characteristic of AKI, and tested them in models of acute and chronic kidney injury to assess therapeutic benefit. We assessed the renoprotective effects of colchicine or metformin in C57BL/6 mice challenged with renal ischemia reperfusion injury (IRI), treated before or after injury. All animals underwent analysis of renal function and biomolecular phenotyping at 24 h, 48 h and 4 weeks after injury. Murine renal tubular epithelial cells were studied in response to in vitro mimics of IRI. Pre-emptive treatment with colchicine or metformin protected against AKI, with lower serum creatinine, improved histological changes and decreased TUNEL staining. Pro-inflammatory cytokine profile and multiple markers of oxidative stress were not substantially different between groups. Metformin augmented expression of multiple autophagic proteins which was reversed by the addition of hydroxychloroquine. Colchicine led to an increase in inflammatory cells within the renal parenchyma. Chronic exposure after acute injury to either therapeutic agent in the context of reduced renal mass did not mitigate the development of fibrosis, with colchicine significantly worsening an ischemic phenotype. These data indicate that colchicine and metformin affect acute and chronic kidney injury differently. This has significant implications for potential drug repurposing, as baseline renal disease must be considered when selecting medication.

## Introduction

Acute kidney injury (AKI) persists as a global public health concern, exerting significant burden on the healthcare system^1,2^. Ischemia–reperfusion injury (IRI) is a major cause of AKI due to complications arising from a range of illnesses and surgical procedures affecting renal blood flow^3^. Renal ischemia occurs during cessation of blood flow with consequent hypoxia, producing structural and functional damage to renal tubular epithelial cells (RTEC) through adenosine triphosphate (ATP) exhaustion and mitochondrial dysfunction^4^. The injury is paradoxically worsened by reperfusion, with the release of pro-inflammatory cytokines, generation of reactive oxygen species (ROS), and upregulation of cell death pathways^5^.

The established concept that AKI reflects a self-limited process accompanied by recovery of function is inaccurate. Observational and experimental studies demonstrate that AKI initiates and contributes to progression of CKD. Patients with AKI have a twofold increased risk of mortality^6^, while survivors have a ninefold increased risk of CKD and a threefold risk for developing end-stage disease^7^. Acute injury can induce renal tubular epithelial cell senescence^8^ and a pro-fibrotic milieu characterized by capillary rarefaction, nephron loss and fibrosis, thereby accelerating the transformation to CKD^9^. Chronic interstitial inflammation is also a feature of CKD, accompanied by progressive cellular infiltration and secretion of associated matrix protein products [e.g. transforming growth factor (TGF)-β, type I collagen]^10^. Despite major advances in understanding the pathophysiology of AKI/CKD, drugs that combat kidney damage remain an unmet need. There are no therapeutic interventions that reliably prevent, treat or improve AKI outcomes^11^: the only treatment for AKI is supportive care^12^.

There is a clear urgency to identify drugs that are effective against AKI/CKD and safe for human use. De novo drug discovery and development is time-consuming and costly, with low rates of successful ‘bench-to-bedside’ transition^11^. Drug repurposing, the re-development of approved drugs in clinical use for different diseases, has emerged as promising in the pursuit for effective prevention of AKI^13^. It offers the opportunity to overcome inherent limitations associated with new drug development and translational capacity, capitalising on the fact that already approved drugs have pre-existing safety, dosing, and toxicity profiles^14^.

Based on our current understanding of AKI and AKI-to-CKD progression, we determined several key pathways as potential targets for drug repurposing studies: oxidative stress, inflammation, and cell death^5,15,16^. We then identified 2 potential drug candidates – colchicine and metformin — that modulate these pathways. Colchicine is used to treat inflammatory conditions, and its beneficial effects have been demonstrated in experimental kidney disease models, including cyclosporine nephrotoxicity^17^ and diabetic nephropathy^18^. Colchicine interferes with microtubule depolymerisation to limit neutrophil adhesion, recruitment and activation. Neutrophils are rapid responders to injury, and their influx into the renal parenchyma is fundamental to AKI pathogenesis. Metformin increases AMP kinase activity and sensitivity to insulin^19^. It ameliorates diabetic nephropathy^20^ and polycystic kidney disease^21^, decreasing oxidative stress within the kidney^22^ and pro-inflammatory mediators^23,24^. We hypothesized that re-purposing colchicine and metformin would be useful in treating AKI as well as progression of AKI-to-CKD, and we investigated whether these drugs have potential beneficial therapeutic implications for patients with concurrent kidney disease and long-term drug exposure.

## Materials and methods

### Reagents

Antibodies against 3-nitrotyrosine (clone 39B6), 4-hydroxynoneal, α-smooth muscle actin (αSMA, clone EPR5368), vimentin (clone EPR3776) and vinculin (clone EPR8185) were from Abcam (Cambridge, UK). Antibodies against Atg5 (clone D5F5U), Atg7 (clone D12B11), Atg12 (clone D88H11), Beclin-1 (clone D40C5), LC3 (clone D3U4C), and β-actin (clone 8H10D10) were from Cell Signalling Technologies (Danvers, MA). Kidney injury molecule (KIM)-1 antibody was from Thermofisher Scientific (Waltham, MA). Colchicine, metformin and hydroxychloroquine were from Sigma Aldrich (St Louis, MO), as was the TUNEL TMR kit. Dihydroethidium was from Molecular Probes (Eugene, USA). Lipopolysaccharide (LPS-EB) was from Invivogen (San Diego, CA). Flow cytometry antibodies CD16/CD32 (2.4G2) purified, CD45 (104) BUV395, CD3 (145-2c11) FITC, NK1.1 (PK136) FITC, CD4 (RM4-5) PE-Cy7, CD8 (53–6.7) APC-Cy7, CD62L (MEL-14) PE, CD44 (IM7) BV711, F4/80 (T45-2342) BV420, CD11b (M1/70) V500, CD11c (HL3) APC, Ly-6G (1A8) PE, CD103 (M290) PE, B220 (RA3-6B2) FITC, were from BD Pharmingen (Franklin Lakes, NJ). TaqMan primers were from Invitrogen (Carlsbad, CA).

### Animals

C57BL/6 mice (wild-type, WT) were from Australian BioResources (ABR, Sydney, Australia). Animals were housed under standard conditions and provided with standard chow ad libitum. All studies were performed using protocols approved by the Western Sydney Local Health District Animal Ethics Committee (#4277) and performed in accordance with the Australian code for the care and use of animals for scientific purposes developed by the National Health and Medical Research Council.

### Renal injury models

Renal IR provides a convenient means of inducing AKI in animals. Twelve-week-old male C57BL/6 mice were anaesthetized using isoflurane and oxygen titrated to effect, with body temperature maintained at 36 °C. For the acute ischemia–reperfusion injury (IRI) model a mid-line abdominal incision was performed and microaneurysm clamps were placed to occlude both renal pedicles for 20 min (to induce ischemia) after which the clamps were removed (reperfusion). The abdomen was closed with 5/0 monofilament suture. Prior to IRI, mice were subjected to intraperitoneal injections of either a vehicle control (PBS), colchicine (0.4 mg/kg, 1 h prior to IRI) or metformin (0.25 mg/kg the day prior and the day of IRI). Drug doses were chosen based on previous methodology described in the literature^17,20,25,26^. In certain experiments, metformin (0.25 mg/kg/day) and hydroxychloroquine (50 mg/kg/day) were administered the day prior and day of IRI. In further experiments, mice underwent renal IRI, followed by administration of a single dose of metformin or colchicine the day following surgery, and were euthanised at 48 h.

For the chronic kidney injury model, mice were challenged with unilateral renal ischemia–reperfusion injury performed only on the left kidney. At 7 days post-reperfusion a right nephrectomy was performed. A right flank incision was made subcostally and the kidney identified. The right renal pedicle was tied with 4/0 silk suture at the hilum and at the origin with the aorta/inferior vena cava. The renal pedicle was cut and the right kidney dissected away from the perirenal fat. The renal bed was observed for hemostasis, and the abdomen subsequently closed with 5/0 monofilament suture. In this manner all renal function is dependent on the healing, injured left kidney, mimicking a clinical scenario of reduced renal mass. Post-nephrectomy, mice were given intraperitoneal injections of either a vehicle control or colchicine (0.4 mg/kg) or metformin (0.25 mg.kg) on alternate days for 4 weeks. At the time of euthanasia (D + 1 for AKI, D + 28 for CKD), blood was collected, kidney tissue was snap frozen, embedded and frozen in optimal cutting temperature (OCT) compound or fixed in 10% neutral buffered formalin.

### Kidney histology

Kidneys embedded in paraffin were sectioned at 4 μm and stained with hematoxylin and eosin (H&E) using standard methods^27^. Briefly, sections were deparaffinised, immersed in xylene, then sequentially placed in 100%, 95%, and 75% ethanol. Slides were stained with Haematoxylin (POCD Healthcare; North Rocks, Australia), washed, then immersed in Eosin (POCD Healthcare). Sections were mounted, coverslipped and imaged using a Nanozoomer Imaging System (Hamamatsu Photonics; Japan). Markers of acute tubular damage (tubular dilatation, cell necrosis, infarction, and cast formation) were scored by semi-quantitative calculation of percentage of the corticomedullary junction that displayed such features: 0, none; 1, 1–10%; 2, 11–25%; 3, 26–45%; 4, 46–75%; 5, > 75%. Histological examination was performed in a ‘blinded’ fashion by 2 independent assessors on 5 randomly selected fields (magnification × 100). Light microscopy images were acquired under identical settings.

Picosirius red staining was also performed according to standard protocol. Sections were deparaffinized and placed in a 0.1% solution of Sirius, washed and cover-slipped with mounting media. Slides were viewed under brightfield conditions. Fibrosis scores were assessed in 5 randomly selected corticomedullary areas and quantified using ImageJ.

### Assessment of renal function

Renal function was determined by measurement of serum creatinine using the Siemens Dimension Vista System.

### Murine RTEC cultures

Primary C57BL/6 renal tubular epithelial cells were isolated as described previously^28^. Kidneys were digested using multi-tissue dissociation kit and GentleMacs (Miltenyi, Bergisch Gladbach, Germany), incubated with CD326 (EpCAM) microbeads (Miltenyi) and passed through LS columns. The positive cell fraction was suspended in defined K1 medium: DMEM/F12 medium supplemented with 25 ng/ml epidermal growth factor (Sigma Aldrich, St Louis, MO), 1 ng/ml prostaglandin E_1_ (Cayman Chemicals, Ann Arbor, MI), 5 × 10^−11^ M triiodothyronine (Sigma Aldrich), 5 × 10^−8^ M hydrocortisone (Sigma-Aldrich), insulin–transferrin–sodium selenite supplement (Sigma Aldrich), 1% penicillin/streptomycin (Thermofisher Scientific, Waltham, MA), 25 mM HEPES (Thermofisher Scientific), and 5% FCS (Thermofisher Scientific) and cultured on collagen-coated dishes (BD Biosciences, Franklin Lakes, NJ).

Cell passages 2–4 were used for experimental work. Primary RTEC cultures were treated for 24 h in the following groups: untreated, LPS only (1 μg/mL, InvivoGen; San Diego, USA), LPS + colchicine (1 μM), LPS + metformin (1 mM). In further experiments, RTEC were subjected to normoxia (FiO_2_ 21%) or hypoxia (FiO_2_ 1%) for 24 h. All drug treatments were administered 2 h prior to LPS stimulation or hypoxia. Cell lysates were collected for RNA and protein.

### Western blot analysis

Tissue or cells were homogenized in cold RIPA buffer (Cell Signaling Technology) that contained protease inhibitor cocktail (Sigma Aldrich) and phosphatase inhibitor cocktail (Roche Applied Science, Hercules, CA). Lysates were quantified using a DC assay (BioRad, Hercules, CA). Protein was resolved by SDS-PAGE and transferred onto nitrocellulose membranes (BioRad). Blots were probed with primary antibodies and visualized on an Odyssey Imaging System (Licor, Lincoln, NE) using ImageStudioLite (Licor). The intensity of the bands was quantified using ImageStudioLite**.**

### RNA extraction and quantification by real-time PCR

RNA was extracted using ISOLATE-II RNA MiniKits (Bioline, London, UK) with on-column DNase treatment. RNA was quantified using a Nanodrop (BioTek, Winooski, VT), and reverse-transcribed using a SensiFAST cDNA synthesis kit (Bioline). cDNA was amplified in triplicate with gene-specific primers (Invitrogen) using a CFX384 real-time PCR machine (BioRad). Thermal cycling conditions were 95 °C for 2 min, followed by 40 cycles of 95 °C for 5 s and 60 °C for 30 s. Data were analysed using the ΔΔCt method with expression normalized to the housekeeping gene and sham-operated animals used as the referent controls.

### Metabolism assay

A Glycolysis Stress Test kit and Seahorse XFe96 Bioanalyzer (Agilent, Santa Clara, CA) was used to measure metabolic flux in real-time. RTEC were seeded in a 24-well V7 PS cell culture microplate (Agilent Technologies; Santa Clara, CA) at a density of 3 × 10^4^ cells per 100 μL per well. Cells were monitored for adherence and left to grow overnight until the desired confluency was reached to obtain a consistent monolayer. Prior to the assay being performed, 1 mM l-glutamine was added to 100 mL of XF DMEM Medium, pH 7.4 (Agilent Technologies) and cells were then washed twice. The plate was placed in a non-CO_2_ incubator at 37 °C for 1 h prior to the assay. Using the Glycolysis Stress Test kit (Agilent Technologies), each of the drug injection ports of the hydrated Sensor Cartridge was then loaded with 10 mM glucose, 1 μM oligomycin (simulant), 50 mM 2-deoxy-D-glucose (2-DG). Basal extracellular acidification rate (ECAR) was measured for 30 min followed by an assessment of glycolytic capacity. Where indicated, cells were treated with LPS (100 ng/ml) and/or metformin (1 mM) for 24 h.

### Flow cytometry

Untreated and colchicine-treated mice were subjected to renal IRI as described. Mice were euthanised and kidneys and spleen harvested. The renal cortex was manually dissected into 1 mm^3^ pieces and digested at 37 °C for 20 min in DMEM/F12 medium (Invitrogen; Carlsbad, CA) with 1 mg/mL type II collagenase (Worthington Biochemicals; Lakewood, NJ), BSA and DNase (Sigma-Aldrich). The spleen was flushed with PBS and manually dissected. The kidney digest and spleen tissue were washed through a 70 μm filter and cell suspensions centrifuged. The cell pellets were resuspended in red blood cell (RBC) lysis buffer, incubated at room temperature for 3 min and washed with MACS buffer (PBS, 0.5% FBS, 2 mM EDTA). Cells were incubated with CD45^+^ MACS microbeads (Miltenyi Biotec; Bergisch, Germany) for 15 min at 4 °C protected from light and then passed through a LS column (Miltenyi Biotec) to obtain CD45^+^ leukocytes. Cells from kidney or spleen were incubated in Fc block (0.5 μg/μL) for 20 min, then stained in antibody cocktails according to the panel of interest for 20 min at 4 °C protected from light, washed with FACS wash buffer (PBS, 2% FCS) and stained with DAPI (viability stain). Acquisition was performed on a BD Fortessa. Where practicable, the same antibody was used in different panels, whereas in others, different fluorochromes were used to minimize spillover spreading to other cell populations. The positive and negative mean fluorescence intensity (MFI) signals for all antibodies were evaluated on murine splenocytes based on FCS versus SSC for calculation of staining index as published previously^29^. Antibody usage for each panel was based on staining index, fluorochrome brightness, antigen density and co-expression and gating strategy. Compensation settings were created using UltraComp eBeads (eBioscience; Massachusetts, USA) for each fluorophore. The compensation matrix was calculated using BD FACSDiva Software. Data were analysed using FlowJo V10 (BD Pharmingen).

### Immunofluorescence

Whole kidneys subjected to renal IRI and snap frozen in OCT were sectioned. Sections were placed on SuperFrost Ultra Plus Adhesion Slides (Thermo Fisher Scientific) and incubated with 10 μM dihydroethidium (DHE) for 22 min at 37 °C in a light-protected, humidified chamber. Sections were washed with PBS, counter-stained with DAPI, and mounted with fluorescent mounting media (DAKO; California, USA). For TUNEL staining, sections were fixed with 4% paraformaldehyde (Sigma-Aldrich) and then permeabilised (0.1% Triton X-100, 0.1% sodium citrate). Sections were washed with PBS and incubated with the reaction mixture containing the TUNEL label and enzyme for 1 h at 37 °C in a light-protected humidified chamber, as per manufacturer instructions. Sections were washed with PBS, incubated with DAPI, and mounted with fluorescent mounting media.

Stained sections were imaged using the Olympus Confocal V1000 with a 40X silicone immersion objective (Olympus, Japan). All images captured were analysed using Olympus FLUOVIEW FV10-ASW Version 4.2b. Cells positive for reactive oxygen species were detected via the Texas Red laser (559 nm), while cell nuclei were identified via the DAPI laser (405 nm). DHE + fluorescence was quantified using ImageJ, where 4 random fields of interest (200 μm × 200 μm) were selected from n = 5 mice and the mean Raw Integrated Density calculated. For TUNEL staining, dead cells were quantified by counting the number of TUNEL-positive cells from 5 randomly selected fields per section, from n = 5 animals.

### Immunohistochemistry

Staining was performed on paraffin-embedded sections (4 μm). Sections were incubated with anti-rabbit Kim-1 antibody (1:200). Immunodetection was performed using the Dako Envision + System-HRP labelled polymer detection kit (Agilent) according to the manufacturer's instructions and slides were counterstained. After mounting, slides were viewed by NanoZoomer (Hamamatsu, Iwata City, Japan). All samples were stained in a single assay. Intensity of staining was calculated using ImageJ.

### Measurement of reactive oxygen species

Kidney tissue was homogenized in ice-cold lysis buffer as published previously^30,31^. Tissue was further lysed by freeze/thaw cycles and passed through a 30-gauge needle. Lysate was centrifuged at 1000* g* (5 min, 4 °C) and then at 28,000* g* (15 min, 4 °C). The supernatant was removed, membranes were resuspended in lysis buffer, and protein concentration was measured using the Bradford microplate method. Superoxide production was initiated by the addition of NADPH and was calculated from the initial linear rate of superoxide dismutase inhibitable cytochrome c reduction using a Biotek Synergy 4 Hybrid Multi-Mode Microplate Reader (Promega, Madison, WI). To determine hydrogen peroxide (H_2_O_2_) activity, kidney tissue was homogenized in ice-cold disruption buffer (containing 0.1 mM EDTA, 10% glycerol, protease inhibitor cocktail, and 0.1 mM phenylmethylsulfonyl fluoride) and further lysed as for superoxide. Lysate was added to the assay mixture and the reaction was initiated by the addition of NADPH. To confirm the H_2_O_2_ signal, catalase was added in parallel wells and the catalase-inhibitable rate of production was quantified from a standard curve.

### Statistical analysis

Data were analysed by Student’s *t*-test, Mann–Whitney *U*-test or ANOVA for multiple group comparisons. A Tukey’s or Dunnett’s multiple comparison post-test was performed where relevant. A *p* value of < 0.05 was assumed to be significant.

## Results

### Short-term exposure to colchicine and metformin protect against AKI

**Figure 1:**
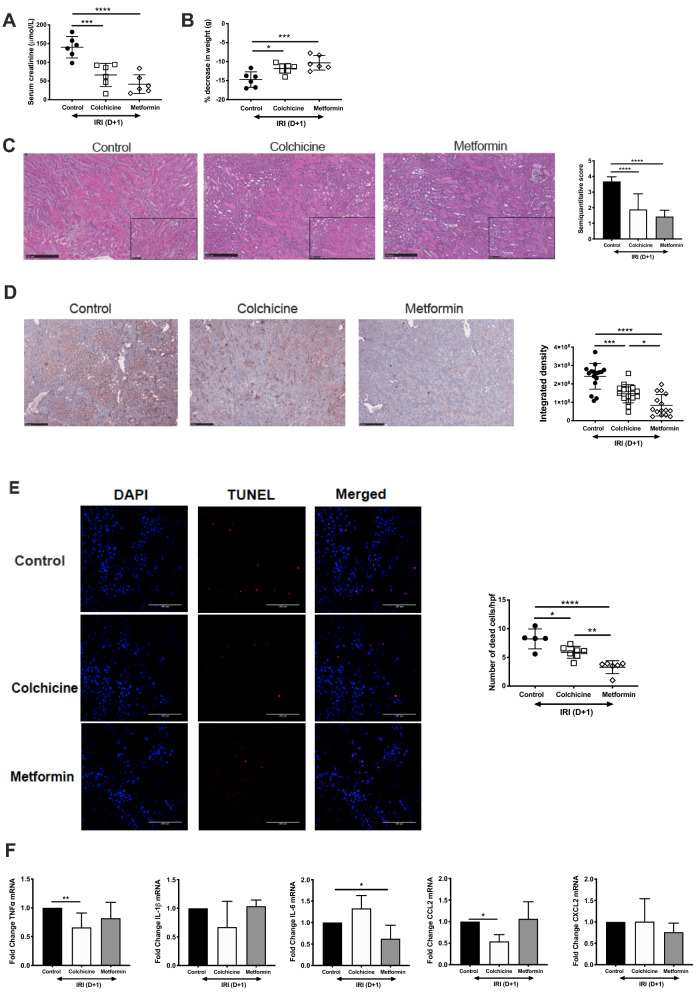
Activated CD47 promotes acute kidney injury. Age-matched male C57BL/6 mice were administered vehicle control (phosphate buffered saline, PBS), colchicine or metformin, and then subjected to bilateral renal ischemia followed by 24 h reperfusion. (**A**) Serum creatinine and (**B**) change in weight were recorded. Kidney tissue was sectioned and stained with (**C**) hematoxylin and eosin and (**D**) KIM-1 with semiquantitative analysis of tissue injury. Representative photomicrographs are shown, original magnification 10 × , scale bar is 250 μm; inset is 20× , scale bar is 100 μm. (**E**) TUNEL staining of kidney tissue sections were imaged by immunofluorescnce. TUNEL + cells (red) were counted from 5 randomly selected fields from n = 5 animals per group. (**F**) Whole kidney homogenate was prepared for qPCR analysis. All samples were collected for RNA isolation, cDNA synthesis and RT-PCR performed for tumor necrosis factor (TNF)-α, interleukin (IL)-1β, IL-6, CCL2, CXCL2. All data are presented as mean ± SD; **p < 0.01, ***p < 0.001, ****p < 0.0001.

Age-matched male C57BL/6 mice were pre-emptively treated with vehicle control (PBS), colchicine or metformin and challenged with IRI. Both pharmacological agents protected against AKI, with lower serum creatinine (Fig. 1A) and less weight loss (Fig. 1B). This correlated with improved histology (Fig. 1C) and immunohistochemical staining of the transmembrane glycoprotein kidney injury molecule-1 (KIM-1, Fig. 1D). TUNEL staining to assess cell death was substantially lower in both colchicine and metformin-treated mice (Fig. 1E). Analysis of mRNA levels of pro-inflammatory cytokines in whole kidney tissue did not demonstrate a consistent anti-inflammatory effect from either drug. Colchicine exposure led to downregulated tumor necrosis factor (TNF)-α and CCL2 mRNA levels, while metformin lowered IL-6 expression (Fig. 1F).

To demonstrate if metformin or colchicine provided renoprotection post-injury, drugs were administered 24 h following IRI and mice were euthanized at 48 h. A single dose of either drug did not protect against AKI (Fig. S1).

### Protection against AKI is not mediated by reduced oxidative stress

**Figure 2:**
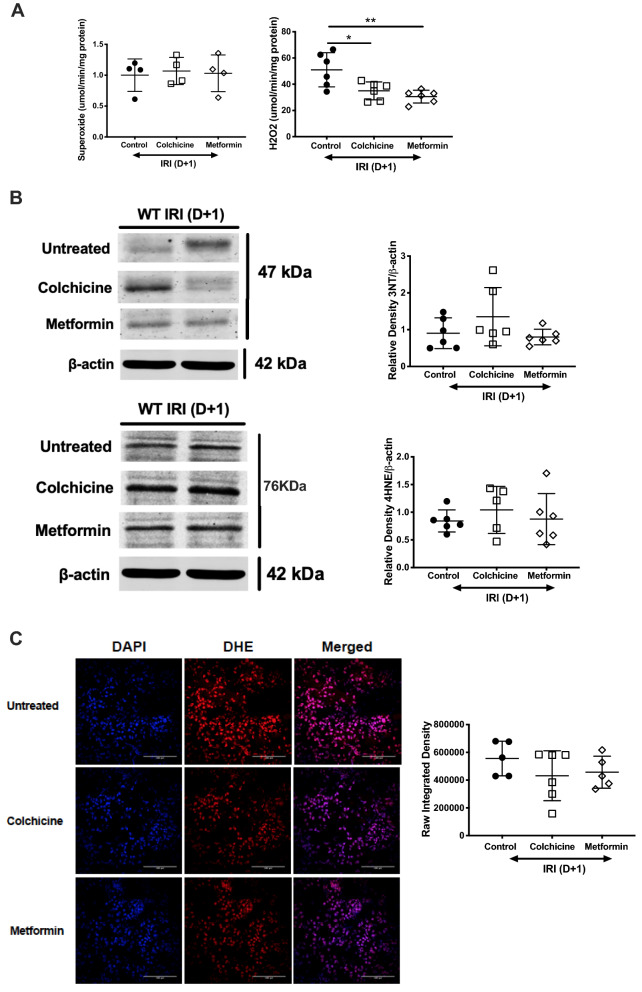
Colchicine and metformin do not limit AKI but altering oxidative stress. Age-matched male C57BL/6 mice were administered vehicle control (phosphate buffered saline, PBS), colchicine or metformin, and then subjected to bilateral renal ischemia followed by 24 h reperfusion. Kidney tissue homogenates were prepared for (**A**) quantification of superoxide and hydrogen peroxide and (**B**) protein was separated by SDS-PAGE to measure ROS-mediated changes. Representative Western blots and combined densitometry for 3-nitrotyrosine and 4-hydroxynoneal is presented as the mean ratio of target protein to β-actin ± SD; *p < 0.05, **p < 0.01, ***p < 0.001. Kidney sections were stained with dihydroethidium (DHE) and imaged by immunofluorescence. (**C**) Representative photomicrographs are shown, original magnification 20 × , scale bar is 100 μm. Intensity of staining was analysed. All data are presented as mean ± SD.

Oxidative stress is a reproducible characteristic of AKI manifested by the generation of reactive oxygen species such as superoxide and hydrogen peroxide^32^. We interrogated the production of both moieties, demonstrating a difference in hydrogen peroxide, but not superoxide, in renal tissue homogenate following treatment with metformin or colchicine (Fig. 2A). Superoxide combines with bioavailable nitric oxide to produce peroxynitrite, and subsequent nitration of tyrosine residues. Lipid peroxidation also ensues, forming electrophilic aldehydes causing direct damage to cell membranes^33^. We assessed protein expression of 3-nitrotyrosine and 4-hydroxynoneal (Fig. 2B) in whole kidney homogenate, and neither downstream event was changed by colchicine or metformin. Oxidative damage was examined further ex vivo through dihydroethidium (DHE) staining. DHE combines with available intracellular superoxide to generate 2-hydroxyethidium that fluoresces red. Kidney sections stained with DHE showed no significant difference in fluorescent intensity, confirming that treatment with either colchicine or metformin prior to renal IRI has no significant effect on the release of ROS (Fig. 2C).

### Metformin protects against AKI via induction of autophagy

**Figure 3:**
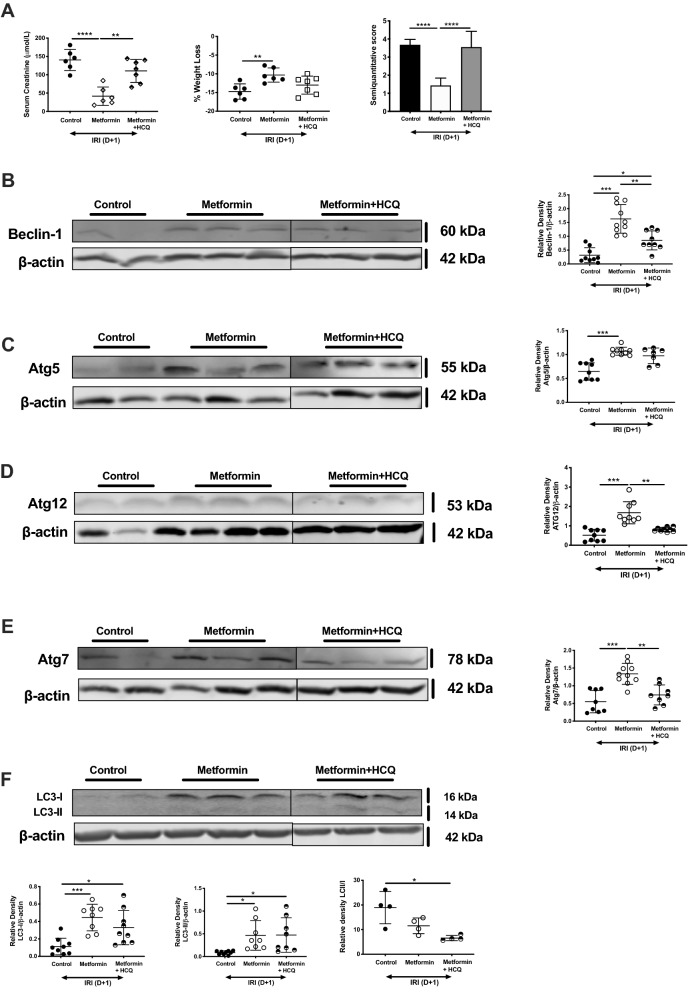
Metformin limits autophagy in acute kidney injury. Age-matched male C57BL/6 mice were administered vehicle control (phosphate buffered saline, PBS), metformin, or metformin and hydroxychloroquine and then subjected to bilateral renal ischemia followed by 24 h reperfusion. (**A**) Serum creatinine, (**B**) change in weight and (**C**) semiquantitative analysis of tissue injury were recorded. Kidney tissue homogenates were prepared and protein separated by SDS-PAGE. Representative Western blots and combined densitometry from groups for (**A**) Beclin-1, (**B**) Atg5, (**C**) Atg12, (**D**) Atg7 and (**E**) LC3I/II from n = 5–7 mice are shown. Densitometry is presented as the mean ratio of target protein to β-actin ± SD; *p < 0.05, **p < 0.01, ***p < 0.001, ****p < 0.0001.

Metformin is used for the treatment of type 2 diabetes mellitus due to its anti-hyperglycaemic actions. However, the mechanism of renoprotection, beyond any effect on glucose, remains unclear in the context of AKI. Autophagy is a multi-step catabolic process involving the degradation of damaged or dysfunctional cytoplasmic components to maintain cellular homeostasis. Upregulation of autophagy has been shown to be protective in AKI^34,35^. Metformin upregulates AMP kinase and downregulate mammalian target of rapamycin (mTOR), both of which can increase autophagy. We investigated whether exposure to metformin augmented autophagic factors following AKI in keeping with a cytoprotective phenotype. Compared to untreated mice, kidney lysate from metformin-treated mice demonstrated significantly increased protein expression of factors involved in multiple stages of autophagy, including Beclin-1*,* Atg5, Atg7, and Atg12 (Fig. 3B–E respectively). There was no significant difference in the expression of LC3-I or LC3-II, but the LC3 II/I ratio was significantly lower in metformin-treated mice (Fig. 3F). To determine whether autophagy is necessary for the renoprotective effect of metformin, we administered the general autophagy inhibitor hydroxychloroquine (HCQ) in conjunction with metformin, measuring the response to AKI and autophagic factor expression. HCQ limits lysosomal acidification and prevents degradation of autophagosomes, and when co-administered with metformin, eliminates protection from AKI. Serum creatinine, changes in weight, and semiquantitative histology scores did not differ from WT control mice (Fig. 3A). We analyzed protein expression of autophagy markers in whole kidney tissue. As expected, combined metformin + HCQ decreased expression of Beclin-1, Atg12, and Atg7 compared to metformin alone. LC3-I and LC3-II levels were unchanged.

### Colchicine changes inflammatory cell influx into the renal parenchyma following AKI

**Figure 4:**
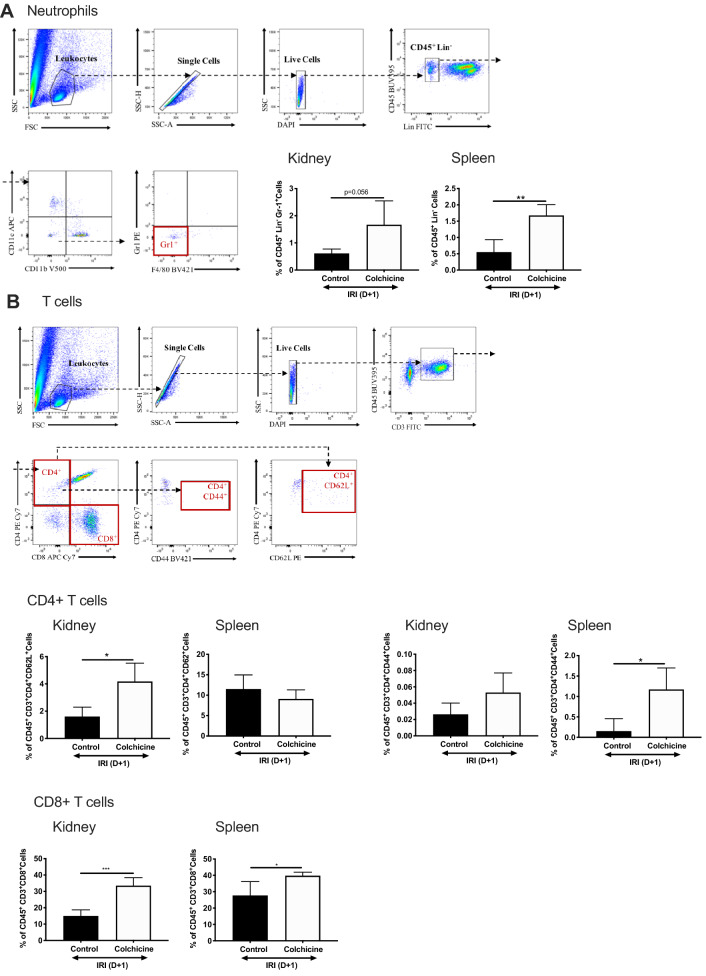

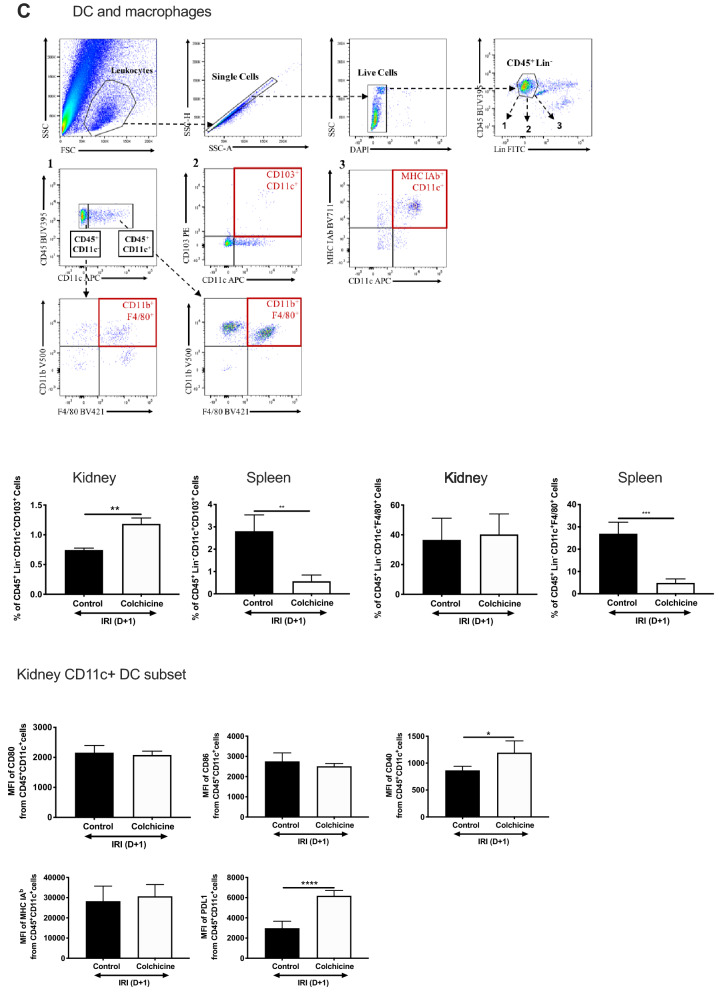
Colchicine increases the inflammatory cell infiltrate within the renal parenchyma. Age-matched male C57BL/6 mice were administered vehicle control (phosphate buffered saline, PBS) or colchicine, and then subjected to bilateral renal ischemia followed by 24 h reperfusion. Kidney and spleen were digested using collagenase, and leukocytes separated from parenchymal cells using CD45^+^ MACS beads. Cells were counted, then stained according to the fluorescence panel required. Gating strategies are shown for each panel, and proportions of cell populations were calculated for (**A**) neutrophils, (**B**) CD4 + CD62L + (naïve) T cells , (**C**) CD4 + CD44 + (activated) T cells, (**D**) CD8 + T cells, (**E**) CD11c + CD103 + DC and (**F**) CD11b + F4/80 + CD11c- macrophages are shown. (**F**) Mean fluorescence intensity (MFI) of cell-surface maturation markers CD80, CD86, CD40, MHC and PDL1 on CD11c + DC isolated from whole kidney. Results are presented as mean ± SD from n = 3 independent experiments, each with n = 2 mice per experiment, *p < 0.05, **p < 0.01, ***p < 0.001.

AKI is associated with the influx of a broad range of pro-inflammatory innate immune cells that promote and (eventually) repair injury. Colchicine treats inflammatory conditions, minimising neutrophil infiltration by inhibiting microtubule depolymerisation^36^. However, the effect of colchicine on neutrophilic and overall inflammatory cell influx into the renal parenchyma following AKI has never been characterised. We generated 4 comprehensive leukocyte profiling panels to assess the major immune cell compartments (and their levels of maturation) involved in AKI: Gr-1^+^ neutrophils, CD4^+^ and CD8^+^ T cells, CD11c^+^ dendritic cells (DC), and CD11b^+^F4/80^+^ macrophages. We assessed cellular infiltrates in both kidney and spleen to determine whether the latter would appropriately reflect changes in inflammatory cell populations within the primary injured organ. The spleen is a reservoir of readily-mobilised cells that comprise the inflammatory infiltrate in AKI, and renal-splenic crosstalk suggests that cellular components may be reflective of those in the kidney^37^. Relevant gating strategies are shown with each leukocyte panel. Unexpectedly, treatment with colchicine led to increased neutrophilic CD45^+^Lin^−^CD11b^−^Gr-1^+^ populations in both kidney and spleen (Fig. 4A). CD4^+^CD62L^+^ T cells were increased only in the kidney (Fig. 4B). CD62L distinguishes naïve T cells and also plays a crucial role in lymphocyte homing to sites of inflammation^38^. CD4^+^CD44^+^ T cells were increased in both the kidney and spleen (only significantly in the latter). CD44 is upregulated after activation of naïve T cells. CD8^+^ T cells were increased in both kidney and spleen.

The heterogeneous mononuclear phagocytic system, comprising macrophage/monocytes and DC, have important sentinel roles orchestrating inflammation related to AKI. The proportion of CD11^+^ DC (gated on CD45^+^Lin^−^ cells) and their maturation status (CD80, CD86, CD40 expression) was unchanged in the kidney regardless of treatment, however CD11c^+^103^+^ DC, which have been implicated in acute tubular injury^39^, was increased in colchicine-treated kidneys following AKI (Fig. 4C), but decreased in the spleen. CD103^+^ DC display distinct functional activities, including cross-presentation and activation of CD8^+^ T cells. CD11b^+^F4/80^+^ macrophage presence were unchanged in the kidney and significantly lower in the spleen.

### Colchicine, but not metformin, worsens the inflammatory cytokine profile of renal tubular epithelial cells

**Figure 5:**
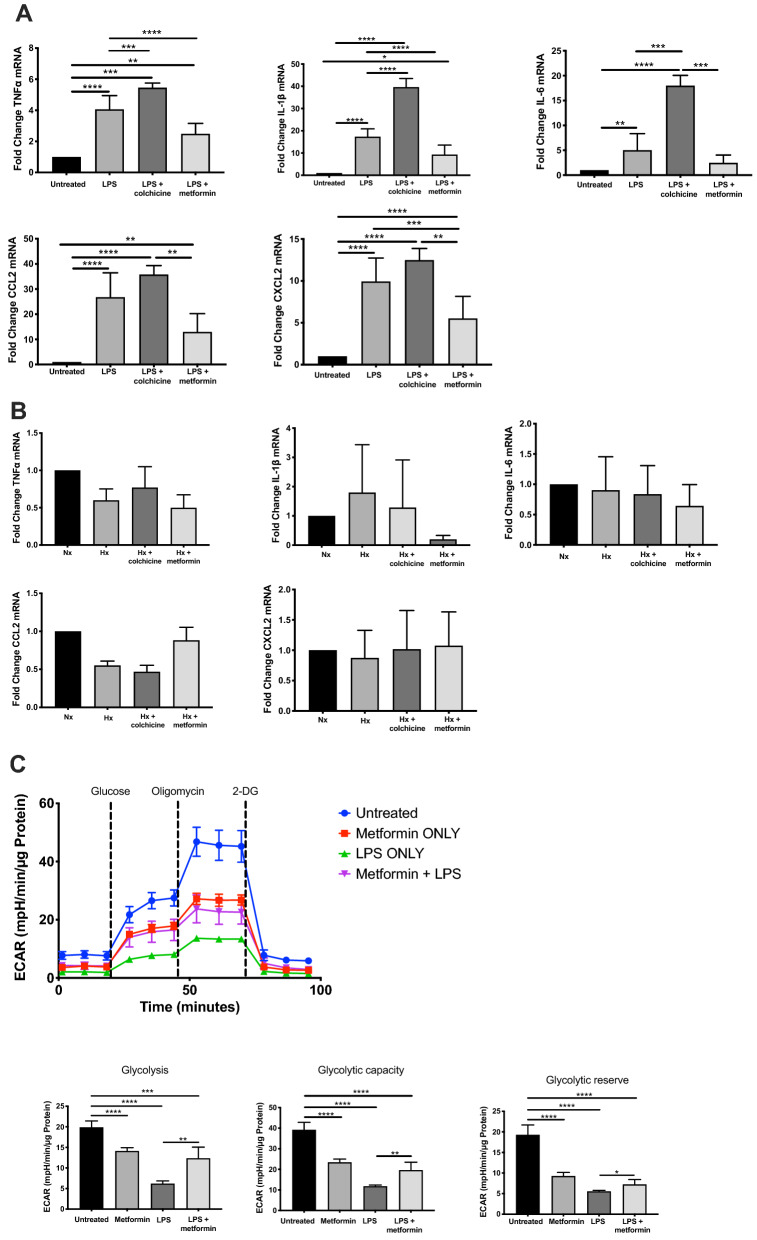
Renal tubular epithelial cell inflammatory and glycolytic responses to exogenous stimuli are mitigated by metformin but not colchicine. Primary renal tubular epithelial cells (RTEC) were isolated from C57BL/6 mice and grown to 70% confluence. Cells were then exposed to (**A**) lipopolysaccharide (100 ng/ml) versus vehicle control (PBS), or (**B**) normoxia (FiO_2_ 21%) versus hypoxia (FiO_2_ 1%) for 24 h. Colchicine (1 μM) or metformin (1 mM) was added to culture 1 h prior to the exogenous stimulus. All samples were collected for RNA isolation, cDNA synthesis and RT-PCR performed for TNF-α, IL-1β, IL-6, CCL2 or CXCL2. All qPCR was run in triplicate and results are presented as fold change ± SD from n = 8 independent experiments. (**C**) SeaHorse XFe96 Bioanalyzer for metabolic flux in RTEC was performed in real-time with/without LPS and metformin. (1) Representative glycolysis stress test showing extracellular acidification rate (ECAR) and (2) calculated glycolysis, glycolytic capacity and glycolytic reserve rates from n = 5 independent experiments. *p < 0.05, **p < 0.01, ***p < 0.001, ****p < 0.0001.

Renal tubular epithelial cells (RTEC) are the primary cellular target of injury in AKI. Primary cultures of murine RTEC were pre-treated with colchicine or metformin and injury was induced via lipopolysaccharide (LPS) stimulation. qPCR was used to compare pro-inflammatory cytokine/chemokine profile from cell lysates. LPS stimulation significantly upregulated the expression of all analysed cytokines and chemokines compared to untreated RTEC, and this was further augmented by colchicine (Fig. 5A). In contrast, metformin-treated RTEC exhibited significantly lower TNFα, IL-1β, CCL2 and CXCL2 expression compared to LPS-only and LPS-colchicine groups. We also compared the cytokine profile of RTEC in the context of hypoxia, but failed to show significant changes in inflammatory profile in response to this stimulus (Fig. 5B).

### Metformin changes the glycolytic profile of RTEC

Changes in RTEC mitochondria and metabolic profile following AKI have been shown to be associated with recovery from acute injury^40^. Proximal tubular cells require high levels of ATP for transport function, derived almost exclusively through oxidative phosphorylation (aerobic metabolism). However, renal glucose uptake and lactate production has been reported in rodent models of AKI, suggesting evidence for enhanced glycolysis^41^. We investigated the effect of an acute inflammatory stimulus on RTEC metabolic profile, and assessed glycolysis via extracellular flux, as measured by basal extracellular acidification rate (ECAR). The addition of LPS to murine RTEC significantly reduced glycolysis, glycolytic capacity and reserve compared to untreated cells, which was improved following co-incubation with metformin (Fig. 5C). Interestingly, metformin alone also modified ECAR, although the significance of this is unclear.

### Colchicine and metformin do not protect against AKI-CKD progression

**Figure 6:**
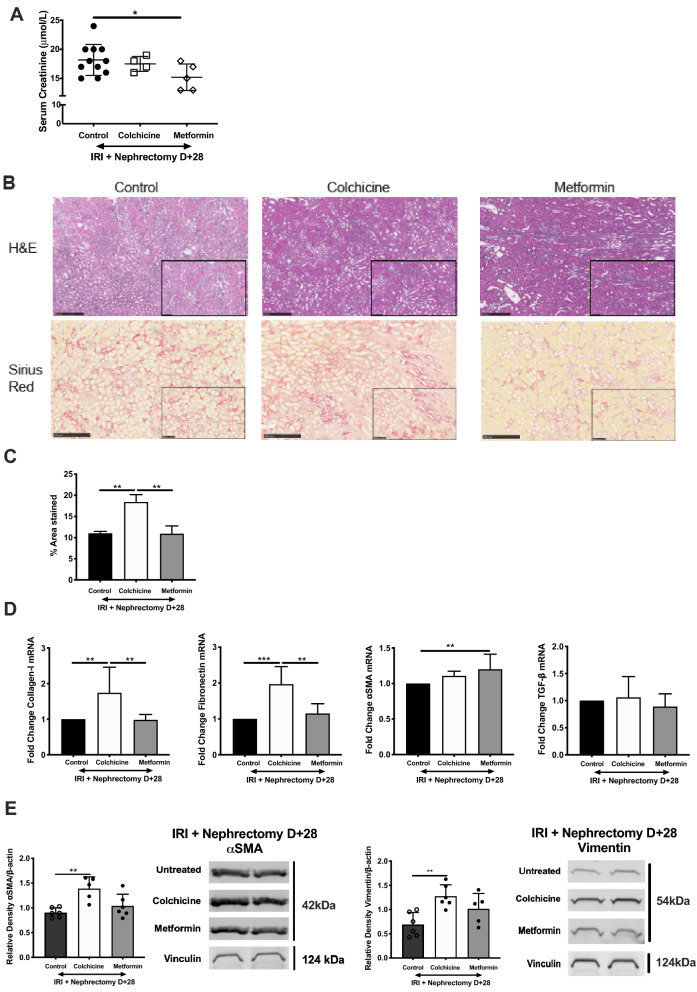
Colchicine worsens development of and metformin fails to modify renal interstitial fibrosis. Age-matched male C57BL/6 mice were subjected to unilateral renal ischemia reperfusion injury + contralateral nephrectomy. Colchicine and metformin were regularly administered from D + 7. At D + 28 post-IRI + Nephrectomy (**A**) serum creatinine was measured. (**B**) Kidney tissues were sectioned and stained with hematoxylin and eosin (H&E) or Sirius red, followed by (**C**) quantitative analysis of fibrosis from n = 6 mice. Whole kidney homogenate was prepared for qPCR analysis. Samples were collected for RNA isolation, cDNA synthesis and RT-PCR performed for (**D**) type I collagen, fibronectin, αSMA and TGF-β. Protein was resolved by SDS-PAGE. (**E**) Quantification of protein expression, in addition to representative western blots for αSMA and vimentin are shown. Densitometry is presented as the mean ratio of target protein to loading control (β-actin or vinculin) ± SD from n = 5–7 samples. PCR was run in triplicate and results are presented as fold change ± SD from n = 6 independent experiments, *p < 0.05, **p < 0.01, ***p < 0.001, ****p < 0.0001.

Current literature has consistently pointed to AKI as an initiating factor in CKD development. A major characteristic of AKI-to-CKD progression is the development of interstitial fibrosis. We have robust evidence for colchicine and metformin successfully treating anticipated AKI, and progressed to assessing the effect of these drugs on preventing the progression of AKI to CKD. Employing a clinically-relevant model of CKD, we performed unilateral ischemia–reperfusion (IR) injury followed by contralateral nephrectomy, reducing renal mass and subsequently relying upon a single injured kidney. Drugs were administered following the resolution of acute injury (D + 7) with regular administration for 4 weeks. This ensured consistency between primary ischemic events, and that the subsequent amelioration of chronic injury was due to the drug alone, rather than mitigating the prior AKI event. Although serum creatinine was marginally improved in metformin-treated mice (Fig. 6A), this was not reflected in histological analysis of tissue sections (Fig. 6B,C). Colchicine promoted collagen deposition compared to control mice, and this was unchanged with metformin. We evaluated transcript and protein expression of fibrotic markers. Type I collagen and fibronectin mRNA levels were significantly increased in colchicine-treated mice (Fig. 6D), as was expression of α-smooth muscle actin (SMA) and vimentin (Fig. 6E).

## Discussion

The absence of agents to prevent or treat kidney injury highlights a crucial area of need as no drug from promising pre-clinical trials have proceeded to routine use. Drug repurposing offers the opportunity to overcome inherent limitations associated with de novo drug development, such as high costs and long development periods^14^. Most importantly, there is greater ease of clinical translation where detailed information is available on pharmacokinetic and pharmacodynamic profiles. In this study we identified and tested 2 drugs commonly used by patients susceptible to acute and chronic kidney injury: colchicine and metformin. Both drugs mitigated injury in a mouse model of AKI when administered pre-emptively, corroborating previous findings that these agents limit injury in other disease models, although we demonstrated differing mechanisms of cytoprotection. We were not able to demonstrate benefit when either drug was administered following AKI, although further testing and dose adjustment may be required. Nevertheless, these results lend support to the repurposing of colchicine and metformin for potential use against AKI in controlled clinical scenarios.

Colchicine is commonly recommended for the treatment of gout, more so in the CKD population in whom non-steroidal anti-inflammatory drugs are contraindicated. Treatment has proven beneficial in a number of non-renal ischemia–reperfusion injury models, such as lung transplantation^25^ and myocardial infarction^42^. To our knowledge, this is the first study to analyse the effect of colchicine in AKI. Although colchicine has demonstrated efficacy in reducing oxidative stress by inhibiting the release of superoxide anion from activated neutrophils^43^, as well as preventing renal cell apoptosis^17,44^, we were not able to replicate either effect.

Colchicine mediates an anti-inflammatory effects by interfering with microtubule polymerisation and neutrophil chemotaxis^11^. Using a comprehensive flow cytometry panel, we show that colchicine treatment immediately prior to renal IRI paradoxically increased neutrophil load within the kidney. Neutrophils are the first innate immune cell responders in IRI, rapidly influxing into the renal parenchyma to release chromatin granule contents, reactive oxygen species and cytokines that perpetuate tissue injury. Neutropenia is protective in cardiac^45^ and hepatic^46^ IRI, and targeting neutrophil recruitment also confers renoprotection^47,48^. We hypothesize that our dosing regimen of colchicine allowed for neutrophilic infiltration into the IRI kidneys, but due to an inhibitory effect on migration, neutrophils were unable to efflux out of the kidneys.

Metformin is frequently used in patients with type 2 diabetes mellitus, and is often preferred to sulphonylurea medication as it does not lead to hypoglycaemia. For the first time we demonstrate that metformin modulates expression of multiple autophagic factors in the renal parenchyma following IRI. Autophagy is a highly conserved cell stress-response pathway, and constitutively required in the kidney, where it is crucial to regulation of RTEC health. Upregulation of autophagic machinery in RTEC limits renal dysfunction and tubular damage in models of renal IRI^34^. Induction of autophagy (Atg) by metformin has been demonstrated in tumorigenesis^49^ but not in the kidney. Here we demonstrate alterations in components of the autophagy pathway, including Beclin-1, and Atg-5, -7 and -12 which are crucial to membrane nucleation and autophagosome elongation and closure, but no significant change in LC3. These effects were limited by the addition of HCQ, supporting the hypothesis that the observed protective effect of metformin requires intact autophagic flux. Metformin limits cell death through modulation of AMPK signalling^50^ and subsequent inhibition of mTOR. Repression of mTORC1 upregulates autophagy and further experimental work will dissect this relationship.

Our data lend support to metformin’s anti-inflammatory effects: metformin-treated post-reperfusion kidneys revealed decreased expression of pro-inflammatory cytokines and chemokines in vivo and in vitro. Metformin treatment significantly reduced RTEC death following IRI, but failed to alter oxidative stress contradicting current evidence supporting its role as a ROS scavenger in the injured kidney^51,52^. We also provide new evidence to demonstrate that metformin regulates RTEC metabolism. Metformin decreased glycolysis, glycolytic capacity and glycolytic reserve. This effect was even more marked following exposure to LPS, although co-incubation with metformin prevented this decrease. RTEC have a high metabolic rate and under homeostatic conditions are reliant on oxidative phosphorylation^53^. Following injury, glycolysis is crucial to maintaining ATP production and the capacity of metformin to preserve glycolysis may account for reduced RTEC death in vivo.

This study recognises the typical clinical presentation of AKI in humans, which is one of established injury. Hence, in addition to testing the prophylactic capability of colchicine or metformin against AKI, we assessed their ability to reduce pre-existing acute injury and used a chronic injury model to evaluate the long-term effectiveness in preventing further renal decline. Prolonged administration of metformin did not mitigate fibrosis and the fibrotic phenotype was worsened by colchicine. The disparity in protection provided by these drugs may lie in the differences in the pathophysiology of acute versus chronic injury within the kidney. While we have demonstrated autophagy to be a key protective pathway upregulated in acute injury^34^, it has not been shown to be integral to the development of chronic injury, minimising the efficacy of metformin. Colchicine was also not beneficial in preventing AKI-to-CKD progression and led to a broad range of inflammatory cells (DC, macrophages, T cells) being retained in the kidney post-reperfusion. Unregulated inflammation is a crucial factor in the transition to CKD, and an increased pro-inflammatory cellular load following prolonged colchicine administration may have contributed to progression of fibrosis. This has important clinical implications as long-term colchicine is being studied in trials to reduce complications following myocardial infarction and in patients with coronary artery disease, which are over-represented conditions in the CKD population.

## Supplementary Information

Supplementary Figure.

Supplementary Legend.
