# Supplementary material for: Repurposing of metformin and colchicine reveals differential modulation of acute and chronic kidney injury
Source: Sci Rep. 2020 Dec 15;10:21968. doi: 10.1038/s41598-020-78936-5 (PMC7738483; doi:10.1038/s41598-020-78936-5)

Title: Repurposing of metformin and colchicine reveals differential modulation of acute and chronic kidney injury.

Running title: Drug repurposing for acute and chronic kidney injury.

Maryam El-Rashid, Danny Nguyen-Ngo, Nikita Minhas, Daniel N. Meijles, Jennifer Li, Kedar Ghimire, Sohel Julovi, Natasha M. Rogers

Supplementary Figure 1

A

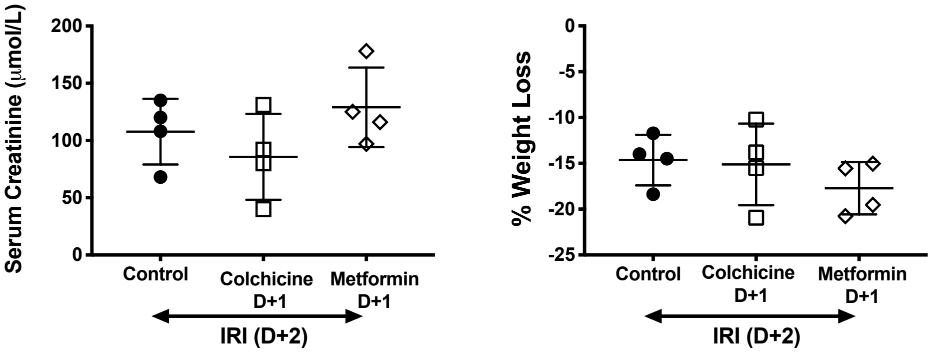

B

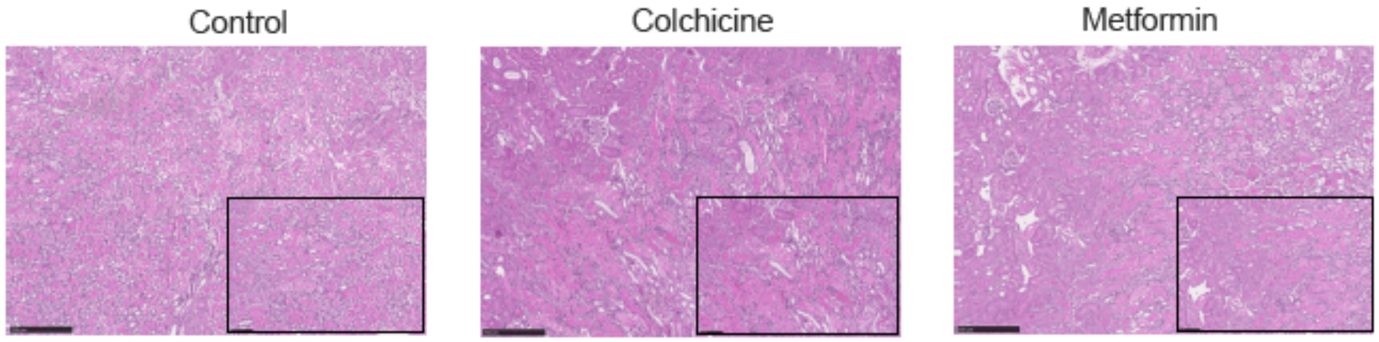

Supplement: Supplementary file 1 — Supplementary Figure. [file 41598_2020_78936_MOESM1_ESM.pdf]
