# Supplementary material for: Repurposing of metformin and colchicine reveals differential modulation of acute and chronic kidney injury
Source: Sci Rep. 2020 Dec 15;10:21968. doi: 10.1038/s41598-020-78936-5 (PMC7738483; doi:10.1038/s41598-020-78936-5)
Supplement: Supplementary file 2 — Supplementary Legend. [file 41598_2020_78936_MOESM2_ESM.docx]

**Title:** Repurposing of metformin and colchicine reveals differential modulation of acute and chronic kidney injury.

**Running title:** Drug repurposing for acute and chronic kidney injury.

Maryam El-Rashid, Danny Nguyen-Ngo, Nikita Minhas, Daniel N. Meijles, Jennifer Li, Kedar Ghimire, Sohel Julovi, Natasha M. Rogers

**Figure legend**

**Supplementary Figure 1**

Age-matched male C57BL/6 mice were subjected to bilateral renal ischemia and then administered vehicle control (phosphate buffered saline, PBS), colchicine or metformin, followed by 48 h reperfusion. (A) Serum creatinine and (B) change in weight were recorded. (C) Kidney tissue was sectioned and stained with hematoxylin and eosin. Representative photomicrographs are shown, original magnification 10x, scale bar is 250μm; inset is 20x, scale bar is 100μm.
